# A guide to selecting high-performing antibodies for CSNK1A1 (UniProt ID: P48729) for use in western blot, immunoprecipitation, and immunofluorescence

**DOI:** 10.12688/f1000research.155928.1

**Published:** 2024-09-13

**Authors:** Riham Ayoubi, Charles Alende, Maryam Fotouhi, Vera Ruíz Moleón, Sara González Bolívar, Kathleen Southern, Carl Laflamme, NeuroSGC/YCharOS/EDDU collaborative group, ABIF consortium

**Affiliations:** 1 Department of Neurology and Neurosurgery, Structural Genomics Consortium, The Montreal Neurological Institute, McGill University, Montreal, Québec, H3A 2B4, Canada

**Keywords:** UniProt ID: P48729, CSNK1A1, Casein kinase I isoform alpha, CK-I alpha, CK1, antibody characterization, antibody validation, western blot, immunoprecipitation, immunofluorescence

## Abstract

CSNK1A1 is a key regulator of various signalling pathways, including the Wnt/β-catenin pathway. Playing a central role in cellular function and disease pathology, CSNK1A1 has emerged as an attractive protein target for therapeutic development. In this study we characterize ten CSNK1A1 commercial antibodies for western blot, immunoprecipitation, and immunofluorescence using a standardized experimental protocol based on comparing read-outs in knockout cell lines and isogenic parental controls. This study is part of a larger, collaborative initiative seeking to address antibody reproducibility issues by characterizing commercially available antibodies for human proteins and publishing the results openly as a resource for the scientific community. While the use of antibodies and protocols vary between laboratories, we encourage readers to use this report as a guide to select the most appropriate antibodies for their specific needs.

## Introduction

A member of the casein kinase family of serine/threonine kinases, Casein kinase I isoform alpha (CSNK1A1), encoded by the *CSNK1A1* gene, acts as a key regulator of various cellular processes such as cell proliferation, apoptosis and key signaling pathways including the Wnt/β-catenin signalling pathway. ^1^^–^^3^ By phosphorylating β-catenin, CSNK1A1 initiates its degradation, acting as a negative regulator of the Wnt signalling pathway. ^4^^,^^5^

Dysregulation of CSNK1A1 activity often leads to various diseased states, including cancer ^6^^,^^7^ and neurodegeneration. ^8^ As such, pharmacological targeting of CSNK1A1 is of significant interest in the research community for clinical intervention. The creation of a publicly accessible database containing trusted antibody characterization data, aiding researchers in assessing antibody suitability, would enable such research.

This research is part of a broader collaborative initiative in which academics, funders and commercial antibody manufacturers are working together to address antibody reproducibility issues by characterizing commercial antibodies for human proteins using standardized protocols, and openly sharing the data. ^9^^–^^11^ Here we evaluated the performance of ten commercial antibodies for CSNK1A1 for use in western blot, immunoprecipitation, and immunofluorescence, enabling biochemical and cellular assessment of CSNK1A1 properties and function. The platform for antibody characterization used to carry out this study was endorsed by a committee of industry academic representatives. It consists of identifying human cell lines with adequate target protein expression and the development/contribution of equivalent knockout (KO) cell lines, followed by antibody characterization procedures using most commercially available antibodies against the corresponding protein. The standardized consensus antibody characterization protocols are openly available on Protocol Exchange (DOI: 10.21203/rs.3.pex-2607/v1). ^12^

The authors do not engage in result analysis or offer explicit antibody recommendations. Our primary aim is to deliver top-tier data to the scientific community, grounded in Open Science principles. This empowers experts to interpret the characterization data independently, enabling them to make informed choices regarding the most suitable antibodies for their specific experimental needs. Guidelines on how to interpret antibody characterization data found in this study are featured on the YCharOS gateway. ^13^

## Results and discussion

**Figure 1.**
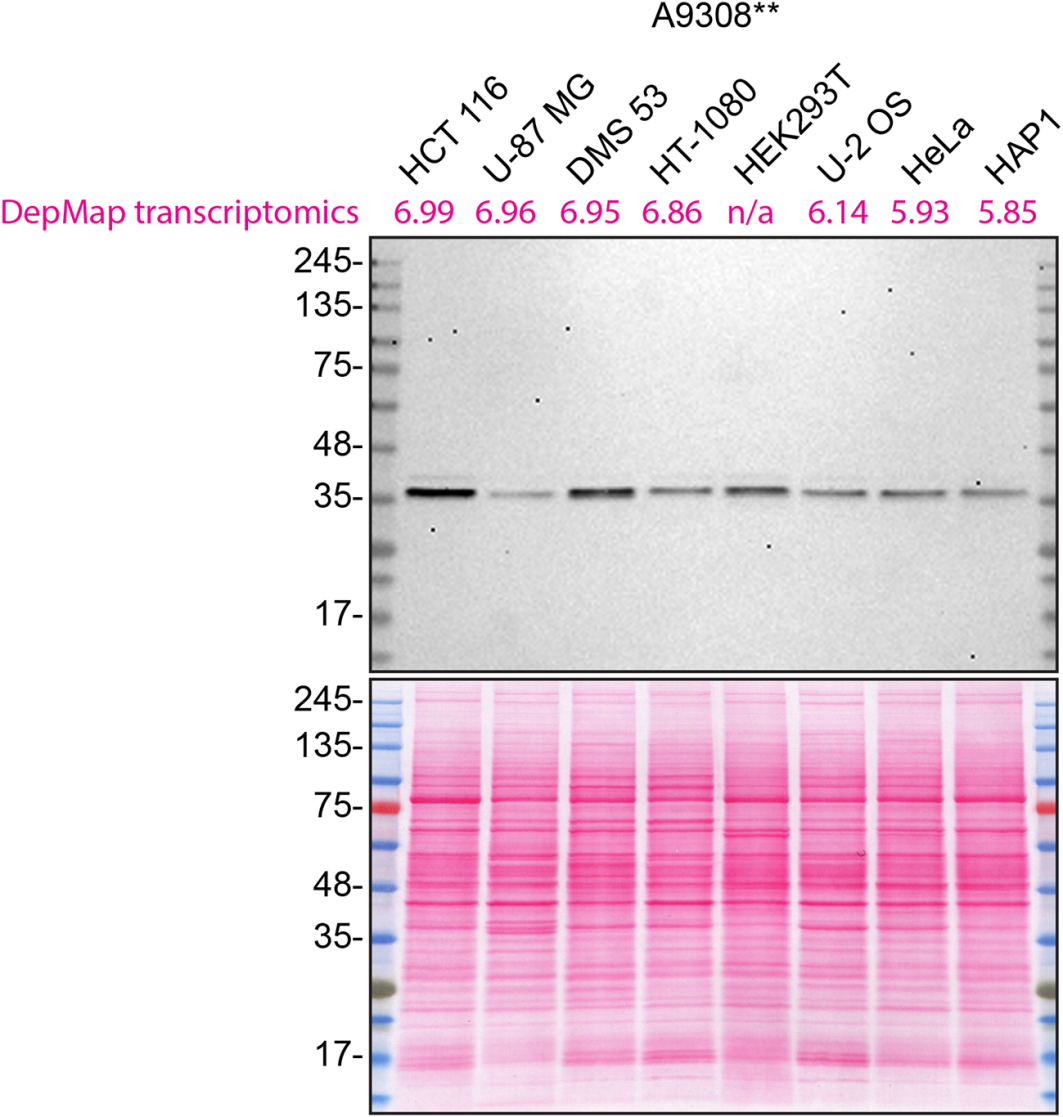
CSNK1A1 western blot on various cell lines. Lysates of HCT 116, U-87 MG, DMS 53, HT-1080, HEK293T, U-2 OS, HeLa and HAP1 were prepared, and 20 μg of protein were processed for western blot with the indicated CSNK1A1 antibody, A9308**, diluted to 1/1000. The Ponceau stained transfer is shown as a loading control.

Our standard protocol involves comparing readouts from WT (wild type) and KO cell lines. ^12^^,^^14^ In the absence of commercially available KO cell lines, siRNA technology can be employed to KD (knockdown) the target gene. ^15^^,^^16^ As *CSNK1A1* is an essential gene in many cancer cells, the application of siRNA is particularly beneficial to maintain the viability of the cells while reducing the expression of the gene, serving as a negative control. ^4^^,^^17^ To determine which cell line demonstrates high expression of CSNK1A1 and thus appropriate for KD, the first step is to identify a cell line(s) that expresses sufficient levels of a given protein to generate a measurable signal using antibodies. To this end, we examined the DepMap (Cancer Dependency Map Portal, RRID:SCR_017655) transcriptomics database to identify cell lines that express the target at levels greater than 2.5 log _2_ (transcripts per million “TPM” + 1), which we have found to be a suitable cut-off. ^9^ To determine which cell line would be appropriate to evaluate CSNK1A1 antibodies, eight cell line backgrounds ( Table 1) were analysed by western blot with three antibodies targeting different epitopes ( Figure 1). The resulting band pattern was analysed across the various cell lines. ^12^ As a result, HCT 116 was identified as the highest expressing cell line as compared to the other cell lines tested here and was thus selected. For simplicity, the western blot with one CSNK1A1 antibody, A9308**, is shown in Figure 1. The cell lines used in Figure 1 as well as the corresponding RNA levels from DepMap are listed in Table 1.

**Table 1.** Summary of the cell lines used.

Institution	Catalog number	RRID (Cellosaurus)	Cell line	Genotype	DepMap transcriptomics log2 (TPM+1)
Abcam	ab255451	CVCL_0291	HCT 116	WT	6.99
ATCC	HTB-14	CVCL_0022	U-87 MG	WT	6.96
ATCC	CRL-2026	CVCL_1177	DMS 53	WT	6.95
ATCC	CCL-121	CVCL_0317	HT-1080	WT	6.86
Abcam	ab255449	CVCL_0063	HEK293T	WT	not available
ATCC	HTB-96	CVCL_0042	U-2 OS	WT	6.14
Abcam	ab255448	CVCL_0030	HeLa	WT	5.93
Horizon Discovery	C631	CVCL_Y019	HAP1	WT	5.85

**Figure 2.**
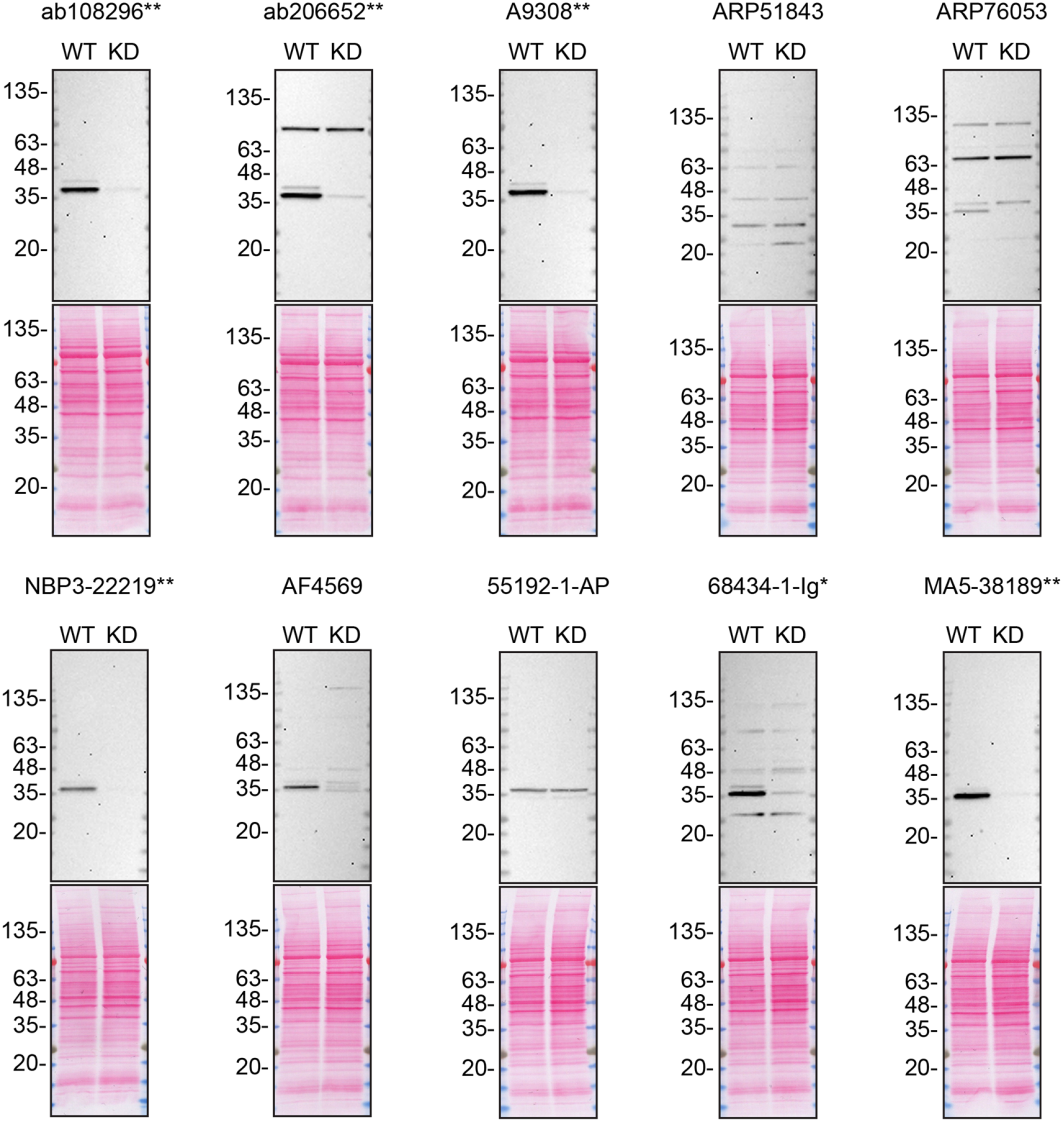
CSNK1A1 antibody screening by western blot. Lysates of HCT 116 WT and *CSNK1A1* KD were prepared, and 50 μg of protein were processed for western blot with the indicated CSNK1A1 antibodies. The Ponceau stained transfers of each blot are presented to show equal loading of WT and KD lysates and protein transfer efficiency from the polyacrylamide gels to the nitrocellulose membrane. Antibody dilutions were chosen according to the recommendations of the antibody supplier. An exception was given for antibody 68434-1-Ig*, which was titrated as the signal was too weak when following the supplier’s recommendations. Antibody dilution used: ab108296** at 1/1000, ab206652** at 1/1000, A9308** at 1/1000, ARP51843 at 1/500, ARP76053 at 1/500, NBP3-22219** at 1/500, AF4569 at 1/100, 55192-1-AP 1/2000, 68434-1-Ig* at 1/3000, MA5-38189** at 1/1000. Predicted band size: 39 kDa. *Monoclonal antibody, **Recombinant antibody.

To screen all ten CSNK1A1 antibodies ( Table 2) by western blot, HCT 116 was modified with siRNA, targeting the corresponding *CSNK1A1* gene. HCT 116 WT and *CSNK1A1* KD protein lysates were ran on SDS-PAGE, transferred onto nitrocellulose membranes, and then probed with the antibodies in parallel ( Figure 2).

**Table 2.** Summary of the CSNK1A1 antibodies tested.

Company	Catalog number	Lot number	RRID (Antibody Registry)	Clonality	Clone ID	Host	Concentration (μg/μl)	Vendors recommended applications
Abcam	ab108296 **	1015306-2	AB_10864123	Recombinant-mono	EPR1961(2)	rabbit	0.35	Wb
Abcam	ab206652 **	1045172-2	AB_2925161	Recombinant-mono	EPR19824	rabbit	0.64	Wb, IP
ABclonal	A9308 **	4000001860	AB_2863708	Recombinant-mono	ARC1860	rabbit	0.80	Wb
Aviva Systems Biology	ARP51843	QC24062-43315	AB_2045492	Polyclonal	-	rabbit	0.50	Wb
Aviva Systems Biology	ARP76053	QC48381-42018	AB_3073938	Polyclonal	-	rabbit	0.50	Wb
Bio-Techne (Novus Biologicals)	NBP3-22219 **	231009	AB_3075892	Recombinant-mono	SR1581	rabbit	n/a	Wb
Bio-Techne (R&D systems)	AF4569	ZSM0120091	AB_2084642	Polyclonal	-	sheep	0.20	Wb
Proteintech	55192-1-AP	00060980	AB_11183034	Polyclonal	-	rabbit	0.45	Wb, IP, IF
Proteintech	68434-1-Ig *	10037603	AB_3073926	Monoclonal	1E2E7	mouse	1.00	Wb
Thermo Fisher Scientific	MA5-38189 **	YJ4090056	AB_2898106	Recombinant-mono	ARC1860	rabbit	0.80	Wb

Wb = western blot; IP = immunoprecipitation; IF = immunofluorescence; n/a = not available.

* Monoclonal antibody.

** Recombinant antibody.

**Figure 3.**
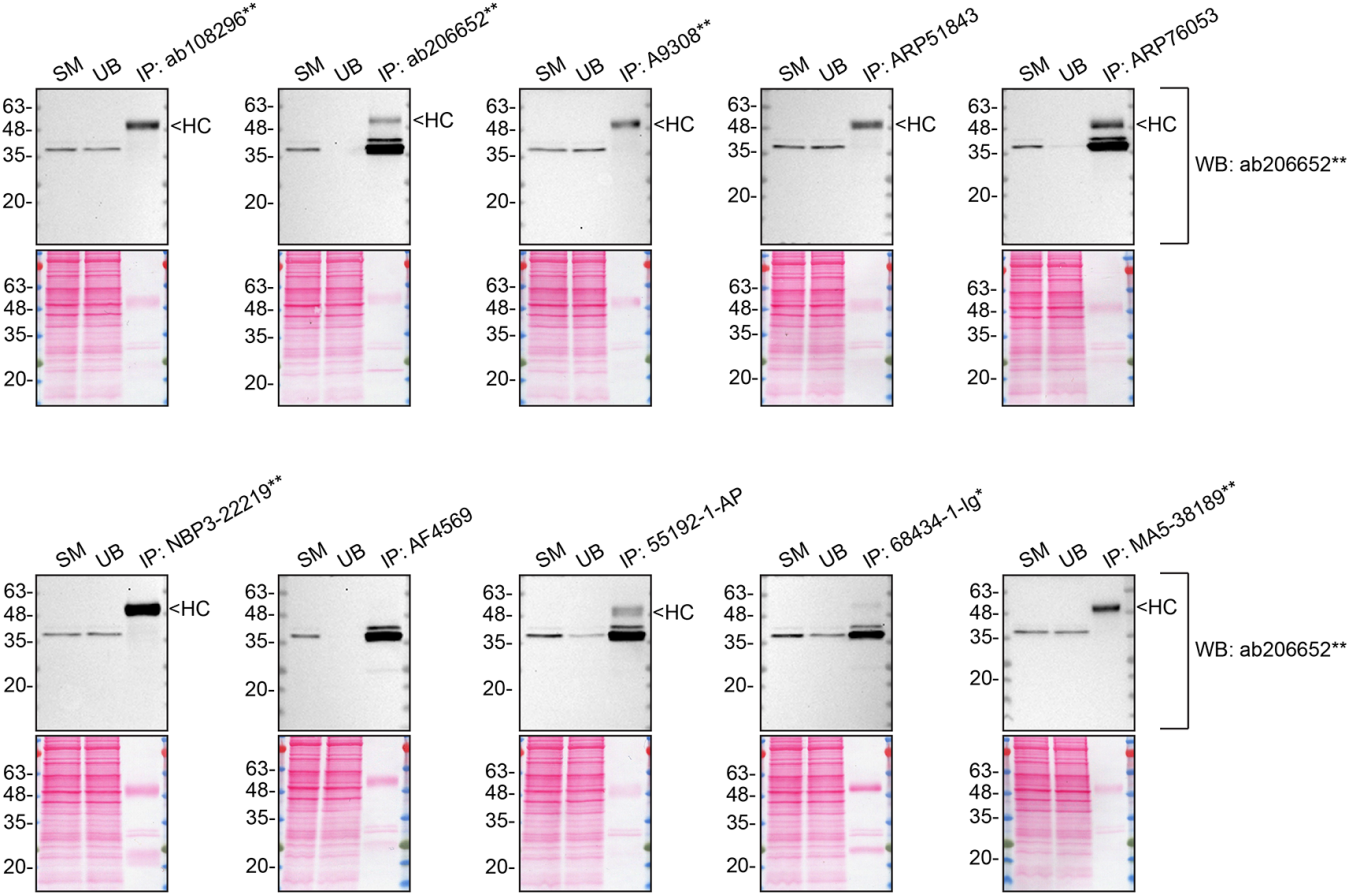
CSNK1A1 antibody screening by immunoprecipitation. HCT 116 lysates were prepared, and immunoprecipitation was performed using 1 mg of lysate and 2.0 μg of the indicated CSNK1A1 antibodies pre-coupled to Dynabeads protein A or protein G. Samples were washed and processed for western blot with the indicated CSNK1A1 antibody. For western blot, ab206652** was used at 1/1000. The Ponceau stained transfers of each blot are shown. SM=4% starting material; UB = 4% unbound fraction; IP = immunoprecipitate, HC = antibody heavy chain. *Monoclonal antibody, **Recombinant antibody.

We then assessed the capability of all ten antibodies to capture CSNK1A1 from HCT 116 protein extracts using immunoprecipitation techniques, followed by western blot analysis. For the immunoblot step, a specific CSNK1A1 antibody identified previously (refer to Figure 2) was selected. Equal amounts of the starting material (SM) and unbound fractions (UB), as well as the whole immunoprecipitate (IP) eluates were separated by SDS-PAGE ( Figure 3).

**Figure 4.**
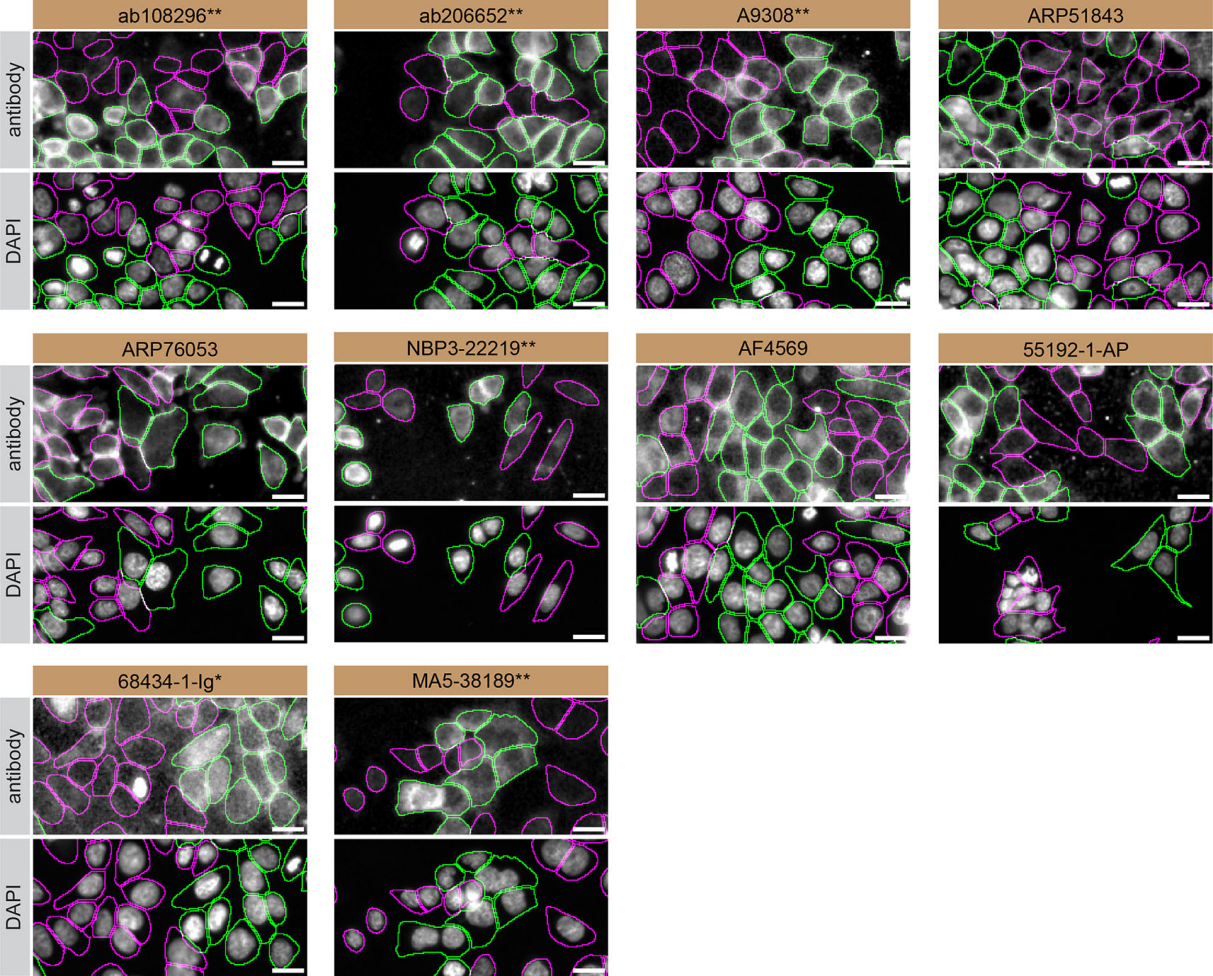
CSNK1A1 antibody screening by immunofluorescence. HCT 116 WT and *CSNK1A1* KD cells were labelled with a green or a far-red fluorescent dye, respectively. WT and KD cells were mixed and plated to a 1:1 ratio in a 96-well plate with optically clear flat-bottom. Cells were stained with the indicated CSNK1A1 antibodies and with the corresponding Alexa-fluor 555 coupled secondary antibody including DAPI. Acquisition of the blue (nucleus-DAPI), green (identification of WT cells), red (antibody staining) and far-red (identification of KD cells) channels was performed. Representative images of the merged blue and red (grayscale) channels are shown. WT and KO cells are outlined with green and magenta dashed line, respectively. When an antibody was recommended for immunofluorescence by the supplier, we tested it at the recommended dilution. The rest of the antibodies were tested at 1 and 2 μg/ml and the final concentration was selected based on the detection range of the microscope used and a quantitative analysis not shown here. Antibody dilution used: ab108296** at 1/350, ab206652** at 1/600, A9308** at 1/800, ARP51843 at 1/250, ARP76053 at 1/250, NBP3-22219** at 1/500, AF4569 at 1/100, 55192-1-AP 1/400, 68434-1-Ig* at 1/500, MA5-38189** at 1/800. Bars = 10 μm. *Monoclonal antibody, **Recombinant antibody.

For immunofluorescence, the ten antibodies were screened using a mosaic strategy. First, HCT 116 WT and *CSNK1A1* KD cells were labelled with different fluorescent dyes in order to distinguish the two cell lines, and the CSNK1A1 antibodies were evaluated. Both WT and KD lines imaged in the same field of view to reduce staining, imaging and image analysis bias ( Figure 4). Quantification of immunofluorescence intensity in hundreds of WT and KD cells was performed for each antibody tested, and the images presented in Figure 4 are representative of this analysis. ^12^

In conclusion, we have screened ten CSNK1A1 commercial antibodies by western blot, immunoprecipitation, and immunofluorescence by comparing the signal produced by the antibodies in human HCT 116 WT and *CSNK1A1* KD cells. To assist viewers in interpreting antibody performance, Table 3 outlines various scenarios in which antibodies may perform in all three applications. Several high-quality and renewable antibodies that successfully detect CSNK1A1 were identified in all applications. Researchers who wish to study CSNK1A1 in a different species are encouraged to select high-quality antibodies, based on the results of this study, and investigate the predicted species reactivity of the manufacturer before extending their research.

**Table 3.** Illustrations to assess antibody performance in western blot, immunoprecipitation and immunofluorescence.

Western blot	Immunoprecipitation	Immunofluorescence
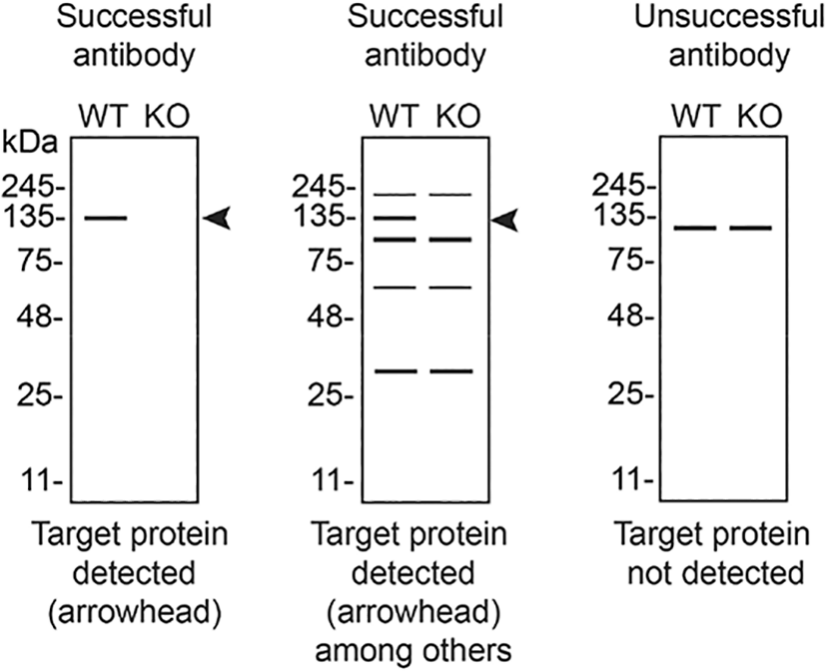	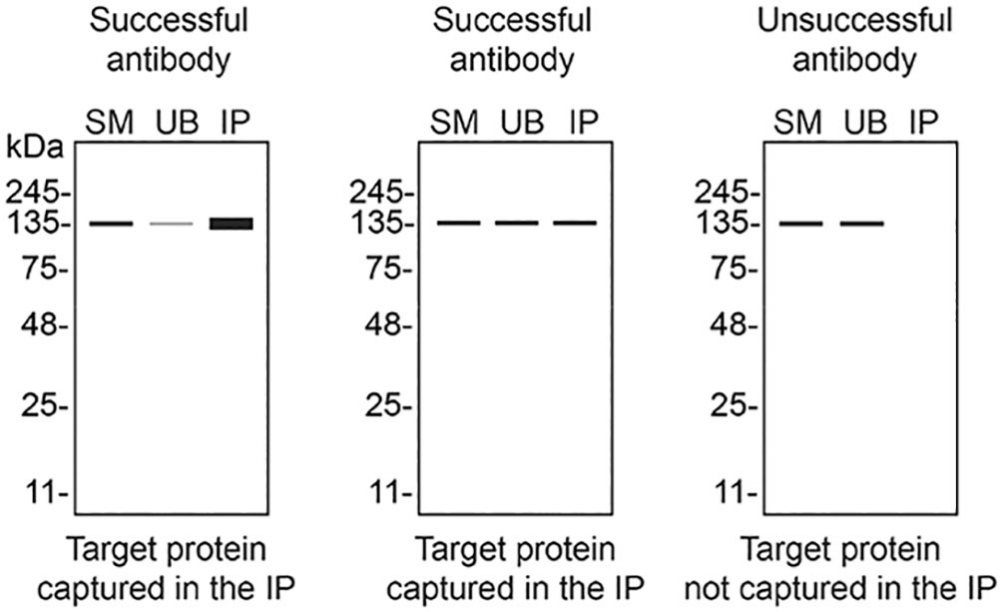	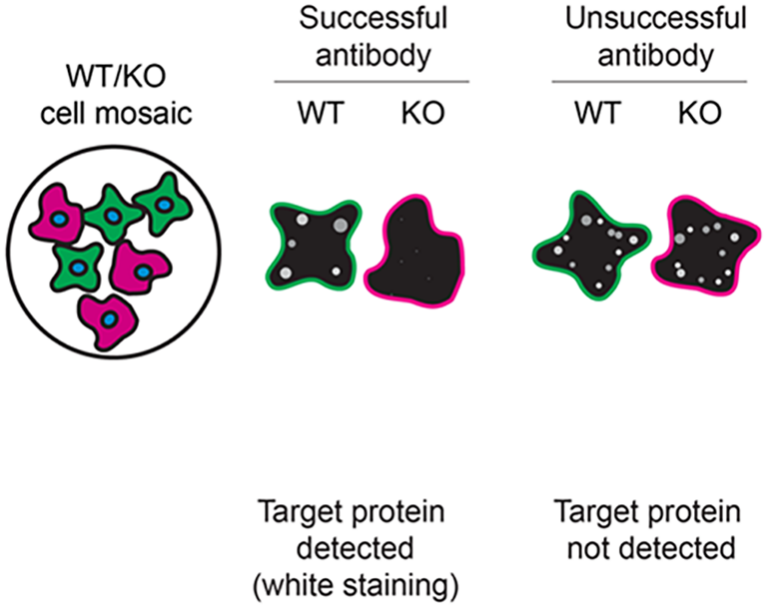

This figure was reproduced with permission from Ayoubi et al., Elife, 2023. ^9^

The underlying data for this study can be found on Zenodo, an open-access repository for which YCharOS has its own collection of antibody characterization reports. ^18^^,^^19^

### Limitations

Inherent limitations are associated with the antibody characterization platform used in this study. Firstly, the YCharOS project focuses on renewable (recombinant and monoclonal) antibodies and does not test all commercially available CSNK1A1 antibodies. YCharOS partners provide approximately 80% of all renewable antibodies, but some top-cited polyclonal antibodies may not be available through these partners.

Secondly, the YCharOS effort employs a non-biased approach that is agnostic to the protein for which antibodies have been characterized. The aim is to provide objective data on antibody performance without preconceived notions about how antibodies should perform or the molecular weight that should be observed in western blot. As the authors are not experts in CSNK1A1, only a brief overview of the protein’s function and its relevance in disease is provided. CSNK1A1 experts are responsible for analyzing and interpreting observed banding patterns in western blots and subcellular localization in immunofluorescence.

Thirdly, YCharOS experiments are not performed in replicates primarily due to the use of multiple antibodies targeting various epitopes. Once a specific antibody is identified, it validates the protein expression of the intended target in the selected cell line, confirms the lack of protein expression in the KO cell line and supports conclusions regarding the specificity of the other antibodies. Moreover, some antibody clones are donated by 2-3 manufacturers (cross-licensed antibodies), effectively serving as replicates and enabling the validation of test reproducibility. All experiments are performed using master mixes, and meticulous attention is paid to sample preparation and experimental execution. In immunofluorescence, the use of two different concentrations serves to evaluate antibody specificity and can aid in assessing assay reliability. In instances where antibodies yield no signal, a repeat experiment is conducted following titration. Additionally, our independent data is performed subsequently to the antibody manufacturers internal validation process, therefore making our characterization process a repeat.

Lastly, as comprehensive and standardized procedures are respected, any conclusions remain confined to the experimental conditions and cell line used for this study. The use of a single cell type for evaluating antibody performance poses as a limitation, as factors such as target protein abundance significantly impact results. ^12^ Additionally, the use of cancer cell lines containing gene mutations poses a potential challenge, as these mutations may be within the epitope coding sequence or other regions of the gene responsible for the intended target. Such alterations can impact the binding affinity of antibodies. This represents an inherent limitation of any approach that employs cancer cell lines.

## Methods

The standardized protocols used to carry out this antibody characterization platform based on comparing readouts in WT and KO cells was established and approved by a collaborative group of academics, industry researchers and antibody manufacturers. The detailed materials and step-by-step protocols used to characterize antibodies in western blot, immunoprecipitation and immunofluorescence are openly available on Protocol Exchange (DOI: 10.21203/rs.3.pex-2607/v1). ^12^ Brief descriptions of the experimental setup used to carry out this study can be found below.

### Cell line and antibodies used

Cell lines used and primary antibodies used in this study are listed in Tables 1 and 2, respectively. To ensure that the cell lines and antibodies are cited properly and can be easily identified, we have included their corresponding Research Resource Identifiers, or RRID. ^20^^,^^21^ HCT 116 cells were cultured in DMEM high glucose (GE Healthcare cat. number SH30081.01) containing 10% fetal bovine serum (Wisent, cat. number 080450), 2 mM L-glutamine (Wisent cat. number 609-065, 100 IU penicillin and 100 μg/ml streptomycin (Wisent cat. number 450201). To KD *CSNK1A1,* the HCT 116 cells were treated twice with 10 nM of *CSNK1A1* SMARTpool siRNA (Horizon Discovery, cat. number L-003957-00-0005). Lipofectamine RNAiMAX (Thermo Fisher Scientific, cat. number 13778030) was used to transfect the siRNA following the manufacturer’s protocol. All other cell types were cultured as recommended by the provider.

Peroxidase-conjugated goat anti-rabbit and anti-mouse (Thermo Fisher Scientific, cat. number 65-6120 and 62-6520), Peroxidase-conjugated donkey anti-sheep antibody (Thermo Fisher Scientific, cat. number A16041) and Peroxidase-conjugated VeriBlot for IP Detection Reagent (Abcam, cat. number ab131366) were used for western blot and immunoprecipitation, respectively. Alexa-555-conjugated goat anti-rabbit and anti-mouse secondary antibodies (Thermo Fisher Scientific, cat. number A-21429 and A-21424) and Alexa-555-conjugated donkey anti-sheep secondary antibody (Thermo Fisher Scientific, cat. number A-21436) were used for immunofluorescence.

### Antibody screening by western blot

HCT 116 WT and *CSNK1A1* KD (listed in Table 1) were collected in RIPA buffer (25 mM Tris-HCl pH 7.6, 150 mM NaCl, 1% NP-40, 1% sodium deoxycholate, 0.1% SDS) (Thermo Fisher Scientific, cat. number 89901) supplemented with 1× protease inhibitor cocktail mix (MilliporeSigma, cat. number P8340). Lysates were sonicated briefly and incubated for 30 min on ice. Lysates were spun at ~110,000 × *g* for 15 min at 4°C and equal protein aliquots of the supernatants were analyzed by SDS-PAGE and western blot. BLUelf prestained protein ladder (GeneDireX, cat. number PM008-0500) was used.

Western blots were performed with precast midi 4-20% Tris-Glycine polyacrylamide gels (Thermo Fisher Scientific, cat. number WXP42012BOX) ran with Tris/Glycine/SDS buffer (Bio-Rad, cat. number 1610772), loaded in Laemmli loading sample buffer (Thermo Fisher Scientific, cat. number AAJ61337AD) and transferred on nitrocellulose membranes. Proteins on the blots were visualized with Ponceau S staining (Thermo Fisher Scientific, cat. number BP103-10) which is scanned to show together with individual western blot. Blots were blocked with 5% milk for 1 hr, and antibodies were incubated overnight at 4°C with 5% milk in TBS with 0,1% Tween 20 (TBST) (Cell Signaling Technology, cat. number 9997). Following three washes with TBST, the peroxidase conjugated secondary antibody was incubated at a dilution of ~0.2 μg/ml in TBST with 5% milk for 1 hr at room temperature followed by three washes with TBST. Membranes were incubated with Pierce ECL (Thermo Fisher Scientific, cat. number 32106) or Clarity Western ECL Substrate (Bio-Rad, cat. number 1705061) prior to detection with the iBright™ CL1500 Imaging System (Thermo Fisher Scientific, cat. number A44240).

### Antibody screening by immunoprecipitation

Antibody-beads conjugates were prepared by adding 2 μg (antibody at known concentration) or 10 μl of NBP3-22219** (antibody at an unknown concentration) to 500 μl of Pierce IP Lysis Buffer (Thermo Fisher Scientific, cat. number 87788) in a microcentrifuge tube, together with 30 μl of Dynabeads protein A - (for rabbit antibodies) or protein G - (for mouse and sheep antibodies) (Thermo Fisher Scientific, cat. number 10002D and 10004D, respectively). Tubes were rocked for ~1 hr at 4°C followed by two washes to remove unbound antibodies.

HCT 116 WT cells were collected in Pierce IP buffer (25 mM Tris-HCl pH 7.4, 150 mM NaCl, 1 mM EDTA, 1% NP-40 and 5% glycerol) supplemented with protease inhibitor. Lysates were rocked for 30 min at 4°C and spun at 110,000 × *g* for 15 min at 4°C. 0.5 ml aliquots at 2.0 mg/ml of lysate were incubated with an antibody-bead conjugate for ~1 hr at 4°C. The unbound fractions were collected, and beads were subsequently washed three times with 1.0 ml of IP lysis buffer and processed for SDS-PAGE and western blot on precast midi 4-20% Tris-Glycine polyacrylamide gels. VeriBlot for IP Detection Reagent:HRP (Abcam, cat. number ab131366) was used as a secondary detection system at a concentration of 0.1 μg/ml.

### Antibody screening by immunofluorescence

HCT 116 WT and *CSNK1A1* KD cells were labelled with a green and a far-red fluorescence dye, respectively (Thermo Fisher Scientific, cat. number C2925 and C34565, respectively). WT and KD cells were plated in a 96-well plate with optically clear flat-bottom (Perkin Elmer, cat. number 6055300) as a mosaic and incubated for 24 hrs in a cell culture incubator, 37°C, 5% CO _2_. Cells were fixed in 4% PFA (in PBS) for 15 min at room temperature and then washed 3 times with PBS. Cells were permeabilized in PBS with 0.1% Triton X-100 for 10 min at room temperature and blocked with PBS with 5% BSA, 5% goat serum and 0.01% Triton X-100 for 30 min at room temperature. Cells were incubated with IF buffer (PBS, 5% BSA, 0,01% Triton X-100) containing the primary CSNK1A1 antibodies overnight at 4°C. Cells were then washed 3 × 10 min with IF buffer and incubated with corresponding Alexa Fluor 555-conjugated secondary antibodies in IF buffer at a dilution of 1.0 μg/ml for 1 hr at room temperature with DAPI. Cells were washed 3 × 10 min with IF buffer and once with PBS.

Images were acquired on an ImageXpress micro confocal high-content microscopy system (Molecular Devices), using a 20× NA 0.95 water immersion objective and scientific CMOS cameras, equipped with 395, 475, 555 and 635 nm solid state LED lights (lumencor Aura III light engine) and bandpass filters to excite DAPI, Cellmask Green, Alexa-555 and Cellmask Red, respectively. Images had pixel sizes of 0.68 × 0.68 microns, and a z-interval of 4 microns. For analysis and visualization, shading correction (shade only) was carried out for all images. Then, maximum intensity projections were generated using 3 z-slices. Segmentation was carried out separately on maximum intensity projections of Cellmask channels using CellPose 1.0, and masks were used to generate outlines and for intensity quantification. ^22^ Figures were assembled with Adobe Illustrator.

## Data Availability

Zenodo: Antibody Characterization Report for CSNK1A1, https://doi.org/10.5281/zenodo.11618594. ^18^ Zenodo: Dataset for the CSNK1A1 antibody screening study, https://doi.org/10.5281/zenodo.13236994. ^19^ Data are available under the terms of the Creative Commons Attribution 4.0 International license (CC-BY 4.0).
