# Supplementary material for: A guide to selecting high-performing antibodies for CSNK1A1 (UniProt ID: P48729) for use in western blot, immunoprecipitation, and immunofluorescence
Source: F1000Res. 2024 Sep 13;13:1055. [Version 1] doi: 10.12688/f1000research.155928.1 (PMC11868743; doi:10.12688/f1000research.155928.1)
Supplement: Peer review history

# Reviewer report: Reviewer response for version 1

**DOI:** 10.5256/f1000research.171163.r337523

**Published:** 2024-11-21

**Authors:** Jan Voskuil

**Affiliations:** 1 Aeonian Biotech Netherlands, Leiden, The Netherlands

Table 3 was not visible, broken images.

 The authors delivered an excellent approach to validate commercial antibodies by comparing their generated signals from a suitable cell line, proven to express the target gene sufficiently for antibody detection, with its cognate knock-down derivative cell line.

 In their approach, they analysed the antibody performance in Western blot (WB), in immuno-precipitation (IP), and in immunocytochemistry (staining of entire cells detected by immunofluorescence, IF).

 Although the objective of the authors is to present their approach as a method of finding high performing antibodies from the market, the data themselves are left without scientific assessment. I think this paper will benefit from an extra paragraph to discuss the quality of the individual data. For example, some antibodies show extra bands in WB, and it is not clear whether these bands would disappear upon further antibody dilution (without loss of specific signal), or that all the bands, the correct and the wrong ones, would equally fade by further antibody dilution. The target CSNK1A1 is located in the cytoskeleton, spindle and cytoplasm. Its location in the IF data is not very clear in many cases. A few sentences on the quality of the data would be desirable and put some extra scientific weight to the article.

 The authors suggest that several antibodies worked well across all three applications, but in my opinion none of them showed proper cellular localisation in the IF data. Table 3 was inaccessible to me.

 This paper is extremely useful for scientists needing a good CSNK1A1 antibody for either WB, or IP. I am not comfortable approving the ICC assessments in this version. I also would like to fully access Table 3.

Are sufficient details of methods and materials provided to allow replication by others?

Yes

Is the rationale for creating the dataset(s) clearly described?

Yes

Are the datasets clearly presented in a useable and accessible format?

Partly

Are the protocols appropriate and is the work technically sound?

Yes

Reviewer Expertise:

Life-time career expertise on antibody characterisation and validation in all immunoassays. Both experience as an academic scientist, and as a commercial provider.

I confirm that I have read this submission and believe that I have an appropriate level of expertise to confirm that it is of an acceptable scientific standard.

# Response

**Published:** 2024-11-29

**Authors:** Carl Laflamme

**Affiliations:** McGill University, Canada

Dear Dr. Voskuil,

 Thank you for taking the time to review our antibody characterization report. We appreciate your feedback.

 We reached out to the F1000 editorial team regarding the issue you flagged with Table 3, and they have resolved it. Thank you for bringing this to our attention.

 While we do not provide assessments of the data in the report, Table 3 includes interpretive tips to guide users. The authors are experts in antibody characterization but not in CSNK1A1 specifically. Therefore, we leave the interpretation of the specific bands detected in western blotting to CSNK1A1 experts. We followed the manufacturers' recommendations for antibody use, and the manufacturers themselves reviewed these data prior to publication. None suggested that titration could improve the signal.

 This report serves to inform researchers about the availability of specific antibodies for immunofluorescence, as demonstrated by the absence of signal in knockout (KO) cell lines for some antibodies. The microscopy parameters used in our antibody screening are intended to assess antibody specificity, rather than to characterize the subcellular localization of CSNK1A1. We welcome CSNK1A1 experts to further investigate the subcellular distribution of this protein. 

 Thank you once again for your comments.

 Best regards,

 Carl Laflamme

# Reviewer report: Reviewer response for version 1

**DOI:** 10.5256/f1000research.171163.r337524

**Published:** 2024-11-09

**Authors:** Deborah Moshinsky

**Affiliations:** 1 Institute for Protein Innovation, Boston, Massachusetts, USA

The authors tested 10 commercially available antibodies targeting CSNK1A1 for activity in Western Blotting, Immunoprecipitation, and Immunofluorescence.  Since a knockout cell line is not available, they compared signals in WT cells to those in which CSNK1A1 was knocked down by siRNA.  

 The Figures clearly allow for data interpretation, and the methods are outlined in enough detail to reproduce the data.  The authors did not interpret the results but gave sufficient justification for not doing so.

 This paper is scientifically sound, and I have no reservations about approving it.

Are sufficient details of methods and materials provided to allow replication by others?

Yes

Is the rationale for creating the dataset(s) clearly described?

Yes

Are the datasets clearly presented in a useable and accessible format?

Yes

Are the protocols appropriate and is the work technically sound?

Yes

Reviewer Expertise:

Antibody characterization and validation

I confirm that I have read this submission and believe that I have an appropriate level of expertise to confirm that it is of an acceptable scientific standard.
